# From isolated openings to streaks: a sequential observational study of loot box openings in livestreamed gaming

**DOI:** 10.3389/fpubh.2026.1858751

**Published:** 2026-07-15

**Authors:** Rodrigo Borja-Block, Florentino Moreno Martín, Antonio Jesús Molina-Fernández, Nerea Cano-Alcalá, Jesús Saiz-Galdós

**Affiliations:** Social and Cultural Determinants of Health Research Group, Department of Social, Work and Differential Psychology, Faculty of Psychology, Universidad Complutense de Madrid, Madrid, Spain

**Keywords:** gambling-like mechanics, livestreaming, loot boxes, monetization cues, public health, streak formation, streamer discourse, verbalizations

## Abstract

Loot boxes have become a major public health concern because they combine chance-based reward structures, repeated purchasing opportunities, and widespread exposure in digital gaming environments, including publicly accessible livestreams. The present study examined how loot box openings were organized, sustained, and terminated in livestreamed contexts, with particular attention to the sequential structure of opening behavior and the verbalizations accompanying it. Using an exploratory-to-analytic sequential observational design with integrated quantitative and qualitative components, we built a broader observational database spanning approximately 65 h and 28 min of streamed material across exploratory and analytic phases. The analyses reported here were based on 13 publicly accessible livestreams from Twitch and YouTube, totaling approximately 27 h and 16 min of video material in the main analytic phase. Across these streams, 490 loot box openings were coded at the level of streams, opening episodes, and individual openings. Results showed that loot box openings were organized predominantly as multi-opening streaks rather than isolated events, with 93.1% of coded openings occurring within streaks. The formation of streaks was more strongly associated with visible monetization-related interface cues, particularly temporary offers and virtual currency, than with broad contextual trigger categories. Once a streak had begun, continuation was the dominant pattern and was not strongly explained by reward rarity, visible affective valence, or near-miss events alone, suggesting a momentum-like sequential process. By contrast, termination appeared more closely associated with disruptions in the ongoing flow of the sequence, including more behaviorally marked moments and some reward outcome categories. Qualitative findings further indicated that streamer verbalizations functioned primarily as evaluative, interpretive, and performative accompaniments to opening activity rather than as reliable mechanisms of self-interruption. Taken together, these findings support a process-based account of livestreamed loot box openings and highlight the importance of sequential organization, monetization salience, and public verbal framing for understanding their potential public health relevance.

## Introduction

Loot boxes have become one of the most debated monetization mechanics in contemporary digital games because they combine uncertain reward delivery, repeated purchasing opportunities, and sustained behavioral engagement within interactive environments. Although they differ from conventional forms of gambling in that their rewards are typically confined to the game environment, a growing body of research has argued that loot boxes are psychologically and structurally akin to gambling due to their reliance on chance-based reward schedules, variable-ratio reinforcement, and the motivational salience of rare outcomes (1–4). These concerns are particularly relevant from a public health perspective because paid loot box systems are widely accessible to adolescents and young adults, have been associated with both gaming- and gambling-related problems, and have prompted increasing regulatory and policy attention across jurisdictions (1, 5–8).

Prior work has identified several reasons why loot boxes warrant close behavioral scrutiny. First, their uncertain and probabilistic structure resembles reinforcement mechanisms long associated with repeated gambling behavior, including reward variability, intermittent reinforcement, and the salience of unpredictable outcomes (2–4). Second, experimental and psychophysiological studies have shown that rare loot box rewards can elicit greater arousal, stronger reward responses, and greater urge to continue opening, suggesting that these events may engage motivational processes comparable to those observed in gambling contexts (2, 3). Third, survey and prospective evidence has linked loot box purchasing to indicators of problem gambling, Internet Gaming Disorder, and Online Gambling Disorder, including in adolescent and young adult samples (1, 5, 6). Taken together, this body of work supports the view that loot boxes are not merely cosmetic or peripheral game features, but monetized chance-based systems with potential relevance for public health.

At the same time, an important limitation of much of the existing literature is that loot box use has often been studied through surveys, experimental manipulations, or analyses of isolated psychological responses to discrete opening events (1–3, 5–7). These approaches have yielded valuable findings, particularly regarding reward reactivity, subjective valuation, problem gambling correlates, and urge following rare outcomes. However, they leave relatively underexplored the ecological organization of loot box opening behavior in real public environments. In particular, the literature has not sufficiently examined whether loot box openings occur primarily as isolated events or as repeated sequences embedded within broader sessions of play, display, and social interaction. This matters because repeated opening behavior may be analytically closer to within-session persistence than to one-off reward responses, and these are not equivalent behavioral phenomena.

This distinction is especially important in light of research on gambling persistence and behavioral addiction processes. Recent work using fine-grained behavioral gambling data has shown that continuation within a session and the probability of quitting may represent distinct dimensions of gambling-related behavior, rather than simple by-products of stake size or reward magnitude alone (9). More broadly, process-based accounts of behavioral addiction emphasize that loss of control and persistence may emerge through dynamic interactions between reward learning, contextual cues, affective regulation, habits, and situational opportunities rather than through isolated events alone (10). In other words, a behavior may become persistent not only because of what was just won or lost, but because it has entered an ongoing sequence that develops its own momentum. This insight has not yet been adequately translated to the study of loot box openings in livestreamed gaming contexts. If loot box openings are similarly organized into repeated sequences, then the relevant unit of analysis may not be the isolated opening, but the streak.

Livestreaming platforms add a further layer of complexity. Loot box openings in streams are not merely private interactions between a player and a game system; they are public, visible, and often explicitly performed for an audience. Recent work on gambling-like elements on Twitch and Kick indicates that video game streams may include enthusiastic responses to paid loot box openings, viewer participation in gambling-like moments, superstitious or magical thinking, and warning talk that coexists with continued exposure to such content (11). These findings suggest that livestreamed loot box openings should not be understood solely in terms of reward mechanics, but also as socially mediated and performative events. In such contexts, opening behavior may be shaped not only by uncertainty and reward salience, but also by visibility, audience feedback, and the dramaturgy of repeated opening as public spectacle.

This performative dimension also has implications for how verbalizations should be understood. Classical work on gambling-related speech has shown that verbal reports produced during gambling-like tasks can reveal evaluative, chance-related, and distortion-linked cognitions, including superstitious beliefs, personal luck attributions, and rationalizations of near misses (12–17). However, this literature has also cautioned that gambling-related verbalizations should not be treated as transparent windows into internal cognition, because what players say may reflect self-presentation, *post hoc* sense-making, or the demands of the task context as much as underlying thought itself (14–16). This caution becomes even more important in livestreaming, where verbalizations are produced in front of an audience and may therefore function simultaneously as evaluation, justification, narration, and performance. As a result, verbalizations in livestreamed loot box openings may help explain not only how players interpret outcomes, but also how opening sequences are maintained, socially framed, or weakly regulated in real time.

Taken together, the literature suggests three major gaps. First, although loot boxes have been widely discussed as gambling-like mechanics and linked to gaming- and gambling-related problems, little is known about how opening behavior is organized sequentially in ecologically valid public contexts. Second, although rare rewards, arousal, and near-miss-like features have received substantial theoretical attention, it remains unclear whether such features actually explain the continuation of repeated opening behavior once a sequence is already underway. Third, although prior studies of gambling-related verbalizations provide a useful conceptual basis, they do not fully account for the public and performative discourse that may accompany loot box openings in livestreams. These gaps matter theoretically because they shape whether loot box openings are best understood as isolated reward events, as within-session streak processes, or as socially performed sequences sustained through a combination of monetization cues, behavioral momentum, and situated verbal framing.

The present study addresses these gaps by examining loot box opening behavior in publicly accessible livestreams on Twitch and YouTube using a sequential observational design developed across exploratory and analytic phases. Rather than treating each opening as an isolated event, the study investigates whether openings are predominantly organized into multi-opening streaks and, if so, how these streaks are formed, sustained, and terminated. In addition, the study examines how streamers' verbalizations accompany these processes, with particular attention to whether such verbalizations function primarily as evaluative and performative accompaniments or as effective markers of self-interruption.

More specifically, the study had four interrelated objectives: to determine whether loot box openings in livestreamed contexts were organized predominantly as isolated episodes or as multi-opening streaks; to examine which contextual and monetization-related cues were associated with the formation of streaks; to analyze which factors were associated with continuation and termination within already established streaks; and to examine how streamer verbalizations accompanied these sequential processes. Accordingly, the study addressed the following research questions: To what extent are loot box openings organized as multi-opening streaks rather than isolated episodes in livestreamed contexts (RQ0)? Which contextual triggers and monetization-related cues are associated with the formation of multi-opening streaks rather than isolated opening episodes (RQ1)? Within already established streaks, which factors are associated with whether an opening is followed by another opening (RQ2)? Within already established streaks, which factors are associated with the termination of the streak across successive openings (RQ3)? And how do streamers' verbalizations accompany the continuation or termination of loot box opening streaks (RQ4)?

Drawing on prior work on loot box reward salience, psychophysiological responses, within-session persistence, process-based accounts of behavioral addiction, and gambling-related verbalizations, the study expected that openings would be organized predominantly within multi-opening streaks rather than as isolated events; that visible monetization-related cues would be more closely associated with streak formation than broad contextual trigger categories; that within-streak continuation would not be explained in a simple or dominant way by rarity, visible valence, or near misses alone; and that verbalizations would function primarily as evaluative, justificatory, and socially performative accompaniments to opening activity rather than as robust mechanisms of stopping. In this way, the study moves beyond an event-based view of loot boxes and toward a process-based account of livestreamed opening behavior.

## Materials and methods

### Study design

This study used an exploratory-to-analytic sequential observational design with integrated quantitative and qualitative components to examine loot box opening behavior in livestreamed gaming contexts (18). Across both phases, the broader observational database encompassed approximately 65 hr and 28 min of streamed material, although the analyses reported in this article are based exclusively on the main analytic phase.

The design included two analytically distinct phases. The first phase was exploratory and aimed to characterize how loot box openings were organized within livestreams, describe the general process of opening loot boxes, identify observable emotional and behavioral correlates of opening events, and detect possible contextual cues associated with the initiation of opening activity. The second phase was analytic and was developed after review of the exploratory observations. Based on those initial findings, a more focused observational matrix was designed to examine the formation, continuation, and termination of opening streaks, as well as the verbalizations accompanying these processes.

This sequential structure was adopted because the study did not begin from a fully stabilized observational framework. Rather, the exploratory phase allowed the research team to become familiar with the phenomenon, identify recurrent patterns in the organization of openings, and refine the coding instrument before conducting the main analytic phase. In particular, the exploratory observations suggested that loot box openings often occurred in close succession and that some contextual interface cues might be associated with the onset of these sequences. These observations motivated the development of a more focused matrix specifically suited to the study of opening streaks.

### Exploratory phase and observer training

The exploratory phase was conducted between October and December 2025. During this stage, seven observers were trained over a three-month period in the use of the observational matrix and in the identification of relevant features of loot box opening behavior in livestreamed environments. This phase involved the review and preliminary coding of approximately 38 hr and 12 min of streamed material.

The purpose of the exploratory phase was not to generate the final analytic dataset reported in this article, but to train observers, stabilize basic observational procedures, and produce broad descriptive observations of the phenomenon. During this phase, the observer team examined how openings were organized within streams, how opening episodes unfolded, which emotional and behavioral reactions accompanied openings, and which contextual triggers or interface cues appeared around them. These preliminary observations were reviewed in January 2026 and informed the refinement of the observational matrix used in the main analytic phase.

### Development of the analytic matrix

Following the exploratory phase, the research team reviewed the preliminary observations and redesigned the coding matrix in January 2026. The refined matrix was intended to capture more precisely the sequential organization of openings, the contextual conditions associated with streak formation, the dynamics of continuation and termination within streaks, and the verbalizations accompanying these processes.

The final matrix included structural variables identifying streams, episodes, and openings; contextual variables related to monetization cues and triggers; outcome-related variables such as reward rarity and reward type; visible affective and emotional variables; and verbalization-related variables, including both the presence and type of verbalization, as well as a quote field for qualitative analysis. The refined matrix was therefore the result of an iterative process combining exploratory observation, theoretical grounding, and instrument development.

The basic unit of recording was the individual opening event, with each row of the matrix corresponding to one loot box opening. Each opening was nested within an opening episode and within a broader livestream session. An opening episode was defined as a context-bound sequence of one or more openings occurring within the same game situation, menu, reward sequence, or opening block. Episodes were classified as isolated episodes when they contained a single opening and as streaks when they contained two or more openings.

Consistent with sequential observational approaches, which emphasize transitions between coded events and the patterned organization of event sequences (19, 20), the ≥2-opening threshold was selected as the minimum point at which opening-to-opening continuity could be observed. Thus, the criterion was not intended to imply strong behavioral persistence by itself, but to distinguish isolated openings from episodes containing at least one observable transition between consecutive openings. To assess the robustness of this operational definition, we also conducted a sensitivity analysis using a stricter ≥3-opening threshold.

Within streaks, continuation referred to openings followed by another opening in the same episode, termination referred to the final opening of the episode, and position within streak referred to the ordinal location of the opening within the sequence.

### Observational matrix and key variables

The refined observational matrix captured, for each individual opening, its position within the stream and episode, relevant contextual and monetization-related cues, outcome characteristics, observable affective reactions, verbalizations, and basic chat indicators.

#### Sequential variables

At the structural level, each opening was assigned an episode opening index indicating its ordinal position within the corresponding opening episode. Continuity flag indicated whether a given opening was followed by another opening within the same episode (1 = followed by another opening, 0 = final opening in the episode). This variable served as a central within-streak outcome in the continuation analyses.

#### Contextual and monetization-related variables

Trigger type captured the proximal situational context surrounding the onset of each opening episode (1 = monetization/shop-related context, 2 = game-directed loot box context, 3 = neutral game play, 4 = social or chat-related triggers, 5 = other neutral triggers). Shop before event indicated whether the in-game shop or monetization interface was visible immediately before the episode (0 = no, 1 = yes). Temporary offers visible coded the presence of explicitly promotional or time-limited offers in the interface (0 = no, 1 = yes). Virtual currency visible coded whether in-game or intermediate currency balances were visibly displayed on screen (0 = no, 1 = yes).

#### Outcome-related variables

Reward rarity indexed the coded rarity of the obtained outcome (1 = common, 2 = rare, 3 = epic, 4 = legendary or highest-tier reward). Reward type captured the main category of the obtained item (1 = cosmetic reward, 2 = performance- or power-related reward, 3 = resources or in-game currencies, 4 = other or mixed types). Near-miss flag recorded whether the opening appeared to involve a near miss or near-miss-like configuration in the reward display (0 = no near miss, 1 = clear near miss, 2 = ambiguous or partial near miss).

#### Affective and behavioral variables

Affective valence coded the overall visible valence of the streamer's immediate reaction (1 = negative, 2 = neutral, 3 = positive). Emotional intensity indexed the observable intensity of that reaction (0 = not observable, 1 = low, 2 = moderate, 3 = high). Observable gesture captured the main visible gesture following the opening (0 = no appreciable reaction, 1 = smile or satisfaction, 2 = annoyance or frustration, 3 = surprise, 4 = other reaction).

#### Verbalization and chat variables

Verbalization present indicated whether a verbalization clearly related to the opening was produced (0 = no, 1 = yes). Verbalization type captured the main discourse function associated with that verbalization (1 = frustration or disappointment, 2 = value or utility evaluation, 3 = luck- or chance-related talk, 4 = rationalization or justification, 5 = money- or cost-related talk, 6 = other discourse functions), with 0 reserved for openings without verbalization. A brief quote field stored short excerpts of the verbalizations to support qualitative analysis, and context notes captured additional contextual information needed to interpret the opening episode. Secondary chat-related indicators included chat activity (overall volume of chat messages), chat valence (predominant emotional tone of the chat), chat mentions loot boxes (whether viewers explicitly encouraged further openings), and streamer interacts chat (whether the streamer interacted with the chat during the episode).

### Stream selection and eligibility criteria

The final analytic sample was drawn from publicly accessible livestreams hosted on Twitch and YouTube. Streams were identified between January and March 2026 through manual searches on the Twitch and YouTube Gaming home interfaces. As a pragmatic criterion for current platform visibility, observers focused on games appearing within the first three rows of the gaming interface at the time of search. Games in which the observed streaming content did not include loot box or randomized reward-opening mechanics relevant to the study were excluded from consideration. From the remaining titles, each observer selected a game with which they were sufficiently familiar to reliably interpret the interface, reward animations, progression systems, and opening-related events.

Within each selected game, observers reviewed available streamers in a language they could understand and transcribe. In practice, the language pool was defined by the observer's ability to reliably code spoken verbalizations and contextual information. Observers then reviewed recent VODs, preferably from the previous 3 days, because archived video allowed pausing, rewinding, and repeated inspection of opening sequences. VODs were screened to determine whether loot box opening activity was clearly observable and whether the surrounding context could be reliably coded.

Streams were eligible for inclusion if they contained clearly observable loot box opening activity, were sufficiently understandable for reliable coding, had adequate visual and audio quality, allowed sequential observation without cuts or interruptions that disrupted the opening process, and remained focused on a single game throughout the relevant session. Streams were excluded if openings were not clearly observable, if the audio or video quality did not allow reliable coding, if the material was too fragmented to reconstruct the sequence, if the streamer changed games during the relevant session, if the content duplicated material already included, or if opening activity appeared commercially forced or externally incentivized through purchase-code promotion or live purchasing activity. Two candidate streams were excluded for this last reason.

If a screened VOD did not contain clear loot box opening activity, observers first reviewed another recent VOD from the same streamer. If no eligible VOD was found, they returned to the streamer list for the selected game and repeated the procedure. The resulting sample should therefore be understood as a purposive convenience sample of publicly available livestreamed loot box opening activity, selected to maximize observability, codability, and ecological relevance rather than statistical representativeness.

### Final sample

The final analytic sample comprised 13 unique livestreams drawn from Twitch and YouTube, totaling approximately 27 hr and 16 min of video material. These streams formed the basis for the main observational coding phase conducted between February and April 2026. Although the broader observational database also included approximately 38 hr and 12 min of material coded during the exploratory phase, the analyses reported in the present study are based exclusively on this main analytic sample.

The final sample included 12 Twitch streams and one YouTube stream. Eleven streams were in Spanish and two were in English. The games represented in the sample were EA Sports FC 26, Brawl Stars, Clash Royale, Counter-Strike, and Pokémon TCG Pocket. Publicly available TwitchTracker metrics indicated substantial variation in the public reach of the included channels (21). Follower counts ranged from approximately 30,000 to 1.3 million, while average live audiences ranged from approximately 150 to 6,000 viewers. These indicators were used only as contextual descriptors of stream visibility and were not included in the inferential analyses. As shown in Table 1, the sample therefore combined variation in platform, language, game title, and public channel reach.

**Table 1:** Characteristics of the final analytic sample.

Characteristic	*n*/value
Total livestreams analyzed	13
Total video material analyzed	27 hr 16 min
Platform: Twitch	12
Platform: YouTube	1
Language: Spanish	11
Language: English	2
EA Sports FC 26	4
Brawl Stars	4
Clash Royale	3
Counter-Strike	1
Pokémon TCG Pocket	1

Streamers were not identified by name in order to reduce unnecessary exposure of individual content creators. Two candidate streams were excluded because the opening activity appeared commercially structured through external purchase-code promotion and live purchasing activity.

### Coding procedure

Streams were viewed and coded using the refined observational matrix. Each coded row corresponded to an individual opening event, while each row was simultaneously nested within a broader episode and within a specific stream. This structure allowed the dataset to represent three analytic levels: the stream level, the episode level, and the opening level. The matrix captured structural identifiers, context-related variables, outcome-related variables, visible affective and behavioral variables, and verbalization-related variables. Quotes and contextual notes were also recorded to support later qualitative analysis. Although the matrix included some auxiliary timing fields, the principal analyses focused on the sequential organization of openings, contextual cues associated with streak formation, continuation and termination dynamics, and accompanying verbalizations.

### Inter-rater agreement and discrepancy resolution

The exploratory training phase was not used as the basis for the formal inter-rater agreement procedure. Instead, agreement was assessed during the main analytic phase. A first full coding version of the analytic matrix was produced during the systematic observation phase, and the principal researcher, who had trained the observer team, subsequently conducted a full recoding of the entire matrix. Inter-rater agreement was calculated on these two coding versions prior to discrepancy resolution.

Agreement was estimated using Cohen's kappa for categorical variables. Kappa values were high across the central variables used in the study, including trigger_type (κ = 0.842), shop_before_event (κ = 0.849), temporary_offers_visible (κ = 0.826), virtual_currency_visible (κ = 0.831), reward_rarity (κ = 0.828), reward_type (κ = 0.841), affective_valence (κ = 0.826), emotional_intensity (κ = 0.846), verbalisation_present (κ = 0.836), and verbalisation_type (κ = 0.800). Agreement was also acceptable for near_miss_flag (κ = 0.794), chat_activity (κ = 0.813), chat_valence (κ = 0.786), chat_mentions_lootboxes (κ = 0.814), and streamer_interacts_chat (κ = 0.811).

For timing-related variables, agreement was assessed using exact agreement and mean absolute difference. Exact agreement was 0.841 for timestamp_hms and 0.855 for latency_seconds, with mean absolute differences of 0.245 s and 0.214 s, respectively.

After reliability had been assessed, discrepancies were reviewed and resolved through discussion, resulting in a consensus-coded dataset. This adjudicated dataset was used for all subsequent analyses. Overall, kappa values were in the substantial to almost perfect range, indicating high coding consistency for the central variables used in the analyses. Because the principal researcher had trained the observer team and later conducted the full recoding, these agreement estimates should be interpreted with some caution, although they still provide a conservative benchmark of coding consistency.

### Quantitative analytic strategy

The quantitative component was organized around the study's main research questions. First, descriptive analyses were used to examine the extent to which loot box openings were organized into streaks rather than isolated episodes. These analyses focused on the number of episodes, the proportion of isolated episodes and streaks, the distribution of openings across them, and the distribution of streak lengths.

Second, streak formation was examined at the episode level by comparing isolated episodes and streaks. These analyses focused particularly on contextual and monetization-related cues observable before the beginning of an opening episode.

Third, within-streak continuation was examined at the opening level, restricted to openings occurring within streaks. These analyses tested whether outcome-related and visible affective variables were associated with whether an opening was followed by another opening.

Fourth, streak termination was examined as a distinct within-streak process. This analysis focused on the factors associated with the closing of an already established streak, rather than on the initial formation of that streak. This analytic logic was consistent with broader work emphasizing within-session continuation and quitting as related but distinct dimensions of gambling-related behavior (9).

Inferential analyses primarily relied on logistic regression models, supplemented by descriptive statistics and contingency tables where appropriate. Standard errors were clustered at the stream level for episode-level models and at the episode level for within-streak opening-level models to account for the nested structure of openings within episodes and streams. Analyses were intentionally kept parsimonious in order to preserve interpretability and avoid overfitting given the size and structure of the dataset. Opening-level models including additional predictors were interpreted as exploratory because the number of termination events was limited relative to the number of parameters estimated.

As a robustness check for the operational definition of streaks, the main episode-level streak formation analysis was repeated using a stricter threshold of three or more openings. This additional analysis examined whether the main conclusions were driven by minimal two-opening episodes or remained stable when streaks were defined as longer repeated sequences. In addition, an exploratory descriptive analysis of temporal density was conducted by estimating streak duration, inter-opening intervals, and the proportion of consecutive opening transitions occurring within 30 and 60 s.

### Qualitative analytic strategy

The qualitative component focused on the verbalizations recorded in the quote field of the analytic matrix and on contextual notes associated with each opening and opening episode. Verbalizations were transcribed literally in the original language of the stream and linked through timestamps to the corresponding opening and/or to the broader opening episode. Verbalizations were coded when they occurred during the opening process or when they explicitly referred to a previous, ongoing, or anticipated loot box opening event. The unit of qualitative coding was the verbal or contextual excerpt situated within a specific opening or episode, rather than the stream as a whole.

The qualitative corpus included 299 coded opening-level excerpts within streaks, including 243 openings with explicit streamer verbalizations and 56 openings with relevant contextual or performative notes but no explicit verbalization. When several verbal excerpts occurred around the same opening, each excerpt was classified according to its dominant discourse function.

A directed qualitative content analysis approach was used, combining deductive and inductive coding. The initial coding framework was informed by prior literature on gambling-related verbalizations and cognitive distortions, as well as by the verbalization categories already included in the observational matrix. These initial categories captured functions such as frustration/disappointment, value or utility evaluation, luck- or chance-related talk, rationalization or justification, money- or cost-related talk, and other or mixed discourse functions.

During repeated review of the verbal and contextual corpus, the coding framework was refined to capture discourse functions that were especially relevant to livestreamed loot box openings. These included social/performance talk, escalation/continuation talk, and stop/limit talk. Social/performance talk captured verbalizations or contextual markers oriented toward viewers or shaped by the performative context of livestreaming. Escalation/continuation talk captured verbal markers that normalized or announced further openings. Stop/limit talk captured statements indicating an intended boundary or stopping point.

Observers initially recorded verbalizations and contextual notes and assigned a verbalization type when identifiable. The principal researcher subsequently reviewed the full corpus, completed missing classifications, and refined borderline cases. Ambiguous excerpts were discussed with observers and members of the research team through an internal auditing and peer-debriefing procedure until a consensual interpretation was reached. The final qualitative classifications were retained in the consensus-coded dataset used for the quantitative and qualitative analyses.

Verbalizations were not interpreted as direct evidence of streamers' internal cognition, but as situated public discourse produced during a livestreamed and performative activity. The qualitative analysis therefore aimed to describe the functions that verbalizations and contextual markers played within opening sequences, including evaluation, justification, dramatization, audience orientation, continuation marking, and occasional limit setting.

### Ethical considerations

This study was reviewed and approved by the Ethics Committee for Research of the Universidad Complutense de Madrid (CEI-UCM; reference no. CE_20250612_18_SOC). The approved research project was titled *Ni Gaming ni Gambling: Riesgos psicológicos y sociales de los juegos de caja de bot*í*n*. The study was based exclusively on publicly accessible livestream content available on open platforms, specifically Twitch and YouTube. No interaction was established with streamers, no intervention was introduced, and no private or restricted material was collected. In accordance with the approved protocol, the research team adopted measures to protect confidentiality and minimize unnecessary exposure, including not foregrounding streamer identities in the analytic reporting, using quotations selectively and only where analytically necessary, and adapting the shared analytic matrix to protect streamer identity.

## Results

### Organization of openings into streaks

To address RQ0, we first examined whether loot box openings in livestreamed contexts were organized primarily as isolated episodes or as multi-opening streaks. Across the final analytic sample, 84 opening episodes were identified. Of these, 34 were isolated episodes containing only one opening, whereas 50 met the operational definition of a streak. At the level of individual openings, the pattern was even clearer: of the 490 coded openings, 456 (93.1%) occurred within streaks, while only 34 (6.9%) occurred as isolated openings. As shown in Table 2, streaks also varied meaningfully in length, with several episodes extending well beyond the minimum two-opening threshold.

**Table 2:** Descriptive structure of the analytic sample.

Variable	*n*	%
Total episodes	84	-
Isolated episodes	34	40.5
Streaks (episodes with ≥ 2 openings)	50	59.5
Total openings	490	-
Openings within streaks	456	93.1
Openings in isolated episodes	34	6.9
Streaks of length 2	15	30.0
Streaks of length 3–5	12	24.0
Streaks of length 6–10	15	30.0
Streaks of length 11+	8	16.0

Percentages for isolated episodes and streaks are calculated over the total number of episodes. Percentages for streak-length categories are calculated over the total number of streaks. Streaks were defined as opening episodes containing at least two openings.

These findings indicate that, in this sample, loot box opening behavior was predominantly organized as a sequential rather than an isolated phenomenon. Although isolated openings were present, the dominant pattern was repeated opening activity clustered within the same episode. Importantly, streaks were not limited to minimal two-opening sequences. A substantial proportion extended into medium-length and longer sequences, suggesting that the observed pattern was not reducible to occasional paired openings, but reflected a broader tendency toward repeated opening once an episode had begun. This emphasis on repeated within-session behavior is consistent with broader gambling research suggesting that continuation and quitting may represent distinct behavioral processes rather than simple extensions of single-event reward responses (9). These findings therefore support treating streaks as the primary sequential unit for the subsequent analyses.

To examine whether this pattern depended on the operational threshold used to define streaks, we conducted a sensitivity analysis using a stricter ≥3-opening criterion. Under this definition, 35 of the 84 episodes (41.7%) were classified as streaks, and 426 of the 490 coded openings (86.9%) still occurred within stricter multi-opening sequences. Episodes containing exactly two openings accounted for 15 episodes and 30 openings, corresponding to only 6.1% of all coded openings. Thus, although the stricter threshold reduced the number of episodes classified as streaks, the substantive conclusion remained unchanged: most openings occurred within repeated sequences rather than as isolated events.

The episode-level model was also re-estimated using the ≥3-opening threshold. The overall pattern remained consistent. Visible virtual currency remained a significant predictor of stricter streak formation (OR = 9.05, 95% CI [1.71, 47.76], *p* =.009). Temporary offers visible retained a positive but non-significant association (OR = 4.26, 95% CI [0.58, 31.19], *p* =.153), while shop before event was not significant (OR = 1.22, 95% CI [0.28, 5.26], *p* =.791). The neutral/other trigger group also showed a positive but non-significant association (OR = 6.07, 95% CI [0.66, 56.10], *p* =.112). These results suggest that the main findings were not an artifact of the original ≥2-opening threshold.

### Temporal density of opening streaks

Because the theoretical relevance of streaks also depends on their temporal density, we conducted an exploratory descriptive analysis of opening pace within streaks. This analysis was informed by gambling research emphasizing event frequency and speed of play as structurally relevant features of gambling products (22). Under the main ≥2-opening threshold, median streak duration was 60 s, although duration varied substantially because livestreamed openings could include skipped animations, commentary, celebrations, pauses, or interaction with the chat.

Inter-opening intervals provided a more direct indicator of opening pace. Across 406 opening-to-opening transitions, the median interval was 14.5 s, and 72.7% of transitions occurred within 30 s. As shown in Table 3, this rapid cadence remained stable under stricter thresholds: when restricted to streaks with ≥3 and ≥5 openings, median inter-opening intervals were 14.0 s and 13.5 s, respectively.

**Table 3:** Temporal density of opening streaks under alternative streak thresholds.

Streak threshold	Streaks	Openings	Opening-to-opening transitions	Median inter-opening interval	Transitions ≤ 30 s	Transitions ≤ 60 s
≥2 openings	50	456	406	14.5 s	72.7%	87.9%
≥3 openings	35	426	391	14.0 s	73.4%	88.2%
≥5 openings	24	386	362	13.5 s	75.1%	89.2%

Inter-opening intervals were calculated from timestamps of consecutive openings within the same streak. The ≥2-opening threshold corresponds to the main operational definition of streaks used in the study; ≥3 and ≥5 thresholds are presented as descriptive robustness checks. Percentages indicate the proportion of opening-to-opening transitions occurring within 30 or 60 s.

Taken together, these findings suggest that many streaks were not only sequentially organized, but also temporally dense. At the same time, these indicators should be interpreted descriptively, because the pace of livestreamed openings can be shaped by game interface animations, skipped animations, streamer commentary, celebration, pauses, and chat interaction.

### Streak formation

To address RQ1, we compared isolated episodes and streaks in relation to contextual triggers and visible monetization-related cues surrounding the onset of opening activity. The descriptive comparison suggested that broad trigger categories were not the clearest differentiator between isolated episodes and streaks. By contrast, specific visible monetization-related cues appeared more informative. As shown in Table 4, streaks were more likely than isolated episodes to occur in the presence of the shop interface, temporary offers, and visible virtual currency.

**Table 4:** Episode-level descriptive comparison of isolated episodes and streaks, including visible monetization-related cues.

Variable	Isolated episodes (*n* = 34), *n* (%)	Streaks (*n* = 50), *n* (%)
Trigger group		
Monetization/Game-directed	31 (91.2)	41 (82.0)
Neutral/Other	3 (8.8)	9 (18.0)
Visible monetization-related cues		
Shop before event	3 (8.8)	27 (54.0)
Temporary offers visible	1 (2.9)	22 (44.0)
Virtual currency visible	2 (5.9)	26 (52.0)

Percentages are calculated within episode type. Streaks were defined as opening episodes containing at least two openings.

This descriptive pattern was reinforced by the episode-level logistic regression models presented in Table 5. In the model including only the broad trigger group, neutral/other triggers were not significantly associated with streak formation. In the fuller model, temporary offers visible (OR = 7.79, 95% CI [1.16, 52.21], *p* =.034) and virtual currency visible (OR = 6.27, 95% CI [1.69, 23.29], *p* =.006) were significantly associated with greater odds of streak formation. By contrast, shop presence alone was not significant once the other monetization-related cues were included.

**Table 5:** Episode-level logistic regression models predicting streak formation from contextual and monetization-related cues.

Predictor	*B*	OR	95% CI for OR	*p*
Model 1			
Neutral/Other trigger group (vs. Monetization/Game-directed)	0.82	2.27	[0.31, 16.66]	0.421
Model 2			
Neutral/Other trigger group (vs. Monetization/Game-directed)	0.50	1.65	[0.24, 11.26]	0.609
Shop before event	−0.10	0.91	[0.18, 4.50]	0.906
Temporary offers visible	2.05	7.79	[1.16, 52.21]	0.034
Virtual currency visible	1.84	6.27	[1.69, 23.29]	0.006

Outcome variable = streak formation (0 = isolated episode, 1 = streak). Odds ratios (OR) are reported with 95% confidence intervals. Model 1 includes only trigger group. Model 2 includes trigger group and visible monetization-related cues. Standard errors were clustered by stream.

These findings suggest that the onset of repeated opening activity was better captured by proximal monetization-related interface cues than by broad contextual trigger categories. In other words, it was not the general situation surrounding the opening that best distinguished streaks from isolated episodes, but the visible presence of concrete monetization features in the interface. This pattern is compatible with research emphasizing the role of reward salience, monetization architecture, and gambling-like design features in shaping risky engagement with loot boxes (1–4, 7).

### Within-streak continuation

To address RQ2, we examined which outcome-related and visible affective variables were associated with whether an opening within an already established streak was followed by another opening. Once a streak had begun, continuation was the dominant outcome. As shown in Table 6, continuation rates were high across most categories, indicating that repeated opening, rather than immediate termination, was the default pattern within ongoing sequences.

**Table 6:** Descriptive continuation rates within streaks by reward rarity, visible affective valence, near-miss status, and related variables.

Variable	Category	*n*	Continuation, *n* (%)	Termination, *n* (%)
Reward rarity	1	216	187 (86.6)	29 (13.4)
	2	104	97 (93.3)	7 (6.7)
	3	83	75 (90.4)	8 (9.6)
	4	34	29 (85.3)	5 (14.7)
Visible affective valence	Negative	80	72 (90.0)	8 (10.0)
	Neutral	269	237 (88.1)	32 (11.9)
	Positive	89	80 (89.9)	9 (10.1)
Near-miss status	No near miss	357	316 (88.5)	41 (11.5)
	Near miss present	56	53 (94.6)	3 (5.4)
	Ambiguous/partial near miss	27	22 (81.5)	5 (18.5)
Visible emotional intensity	Not observable	64	60 (93.8)	4 (6.2)
	Low	213	184 (86.4)	29 (13.6)
	Moderate	143	130 (90.9)	13 (9.1)
	High	16	13 (81.2)	3 (18.8)
Reward type	Type 1	53	50 (94.3)	3 (5.7)
	Type 2	244	226 (92.6)	18 (7.4)
	Type 3	58	39 (67.2)	19 (32.8)
	Type 4	85	76 (89.4)	9 (10.6)

Values are based on the opening-level analytic subset restricted to streaks and valid inferential categories. Percentages are calculated within each category. Continuation indicates that an opening was followed by another opening within the same streak; termination indicates that the opening was the last within the streak.

The regression models presented in Table 7 did not support a simple reward-spike account of within-streak continuation. Reward rarity did not emerge as a robust predictor of continuation across models. Similarly, visible affective valence did not clearly distinguish openings that were followed by another opening from those that were not. Near-miss events also did not provide a strong explanatory account of continuation once the full set of predictors was considered.

**Table 7:** Opening-level logistic regression models predicting within-streak continuation.

Predictor	*B*	OR	95% CI for OR	*p*
Model 1				
Reward rarity 2 (vs. 1)	0.92	2.52	[0.94, 6.72]	0.065
Reward rarity 3 (vs. 1)	0.41	1.51	[0.66, 3.48]	0.331
Reward rarity 4 (vs. 1)	0.07	1.07	[0.40, 2.90]	0.891
Negative valence (vs. neutral)	0.41	1.51	[0.63, 3.61]	0.359
Positive valence (vs. neutral)	0.36	1.43	[0.72, 2.83]	0.309
Low emotional intensity (vs. not observable)	−1.26	0.28	[0.07, 1.08]	0.064
Moderate emotional intensity (vs. not observable)	−0.77	0.46	[0.09, 2.46]	0.368
High emotional intensity (vs. not observable)	−1.79	0.17	[0.02, 1.42]	0.102
Model 2				
Reward rarity 2 (vs. 1)	0.56	1.75	[0.69, 4.45]	0.241
Reward rarity 3 (vs. 1)	0.01	1.01	[0.42, 2.40]	0.989
Reward rarity 4 (vs. 1)	−0.41	0.66	[0.22, 2.01]	0.468
Negative valence (vs. neutral)	0.73	2.08	[0.96, 4.47]	0.062
Positive valence (vs. neutral)	0.56	1.75	[0.72, 4.22]	0.214
Low emotional intensity (vs. not observable)	−1.87	0.15	[0.06, 0.41]	< 0.001
Moderate emotional intensity (vs. not observable)	−1.55	0.21	[0.06, 0.73]	0.014
High emotional intensity (vs. not observable)	−2.59	0.08	[0.01, 0.49]	0.007
Near miss present (vs. no near miss)	1.00	2.72	[0.66, 11.25]	0.167
Ambiguous/partial near miss (vs. no near miss)	0.62	1.86	[0.84, 4.13]	0.128
Reward type 1 (vs. type 2)	−0.75	0.47	[0.09, 2.44]	0.369
Reward type 3 (vs. type 2)	−2.22	0.11	[0.03, 0.35]	< 0.001
Reward type 4 (vs. type 2)	−0.09	0.92	[0.33, 2.58]	0.871

Outcome variable = within-streak continuation (0 = termination, 1 = continuation). Odds ratios (OR) are reported with 95% confidence intervals. Model 1 included reward rarity, visible affective valence, and emotional intensity. Model 2 additionally included near-miss status and reward type. Standard errors were clustered by episode. Model 2 should be interpreted as exploratory given the number of predictors relative to the number of termination events.

In the more complete exploratory model, the clearest effects pointed elsewhere. Lower visible emotional intensity relative to non-observable intensity was associated with lower odds of continuation, and reward type 3 was associated with markedly lower odds of continuation relative to reward type 2 (OR = 0.11, 95% CI [0.03, 0.35], *p* < .001). Overall, these findings suggest that within-streak continuation in this sample was not primarily driven by especially rare outcomes, visibly positive reactions, or near-miss events in a simple or stable way. Instead, once initiated, streaks appeared to continue largely as an ongoing sequential process. This pattern is more consistent with a momentum-like or routinized continuation dynamic than with a model in which continuation depends mainly on isolated high-impact rewards.

### Streak termination

To address RQ3, we examined which factors were associated with the termination of an already established streak across successive openings. Whereas the previous section showed that continuation was the predominant outcome once a streak had begun, the present analyses focused on the conditions under which that ongoing sequence came to an end. As shown in Table 8, termination was unevenly distributed across opening position and outcome categories, but no simple monotonic pattern supported the idea that increasingly rare rewards directly drove closure.

**Table 8:** Descriptive termination patterns within streaks by opening position and key outcome-related variables.

Variable	Category	*n*	Termination, *n* (%)	Continuation, *n* (%)
Opening position within streak	1	47	0 (0.0)	47 (100.0)
	2	49	15 (30.6)	34 (69.4)
	3	35	4 (11.4)	31 (88.6)
	4	28	7 (25.0)	21 (75.0)
	5	23	1 (4.3)	22 (95.7)
	6	23	3 (13.0)	20 (87.0)
	7	19	1 (5.3)	18 (94.7)
	8	18	2 (11.1)	16 (88.9)
	9	16	1 (6.2)	15 (93.8)
	10	14	8 (57.1)	6 (42.9)
	11+	166	7 (4.2)	159 (95.8)
Reward rarity	1	216	29 (13.4)	187 (86.6)
	2	104	7 (6.7)	97 (93.3)
	3	83	8 (9.6)	75 (90.4)
	4	34	5 (14.7)	29 (85.3)
Visible affective valence	Negative	80	8 (10.0)	72 (90.0)
	Neutral	269	32 (11.9)	237 (88.1)
	Positive	89	9 (10.1)	80 (89.9)
Visible emotional intensity	Not observable	64	4 (6.2)	60 (93.8)
	Low	213	29 (13.6)	184 (86.4)
	Moderate	143	13 (9.1)	130 (90.9)
	High	16	3 (18.8)	13 (81.2)
Near-miss status	No near miss	357	41 (11.5)	316 (88.5)
	Near miss present	56	3 (5.4)	53 (94.6)
	Ambiguous/partial near miss	27	5 (18.5)	22 (81.5)
Reward type	Type 1	53	3 (5.7)	50 (94.3)
	Type 2	244	18 (7.4)	226 (92.6)
	Type 3	58	19 (32.8)	39 (67.2)
	Type 4	85	9 (10.6)	76 (89.4)

Values are based on the opening-level analytic subset restricted to streaks and valid inferential categories. Percentages are calculated within each category. Termination indicates that the opening was the final opening within the streak.

The logistic regression models presented in Table 9 reinforced this interpretation. In the parsimonious model, reward rarity category 2 was associated with lower odds of termination relative to rarity category 1 (OR = 0.38, 95% CI [0.16, 0.90], *p* =.028), but this effect did not remain stable in the more exploratory model. By contrast, visible emotional intensity emerged as a more consistent signal of closure. In the exploratory model, low intensity (OR = 5.41, 95% CI [2.10, 13.95], *p* < .001), moderate intensity (OR = 3.81, 95% CI [1.20, 12.15], *p* =.024), and high intensity (OR = 9.81, 95% CI [1.63, 58.95], *p* =.013) were all associated with greater odds of termination relative to non-observable intensity. Reward type 3 also showed a strong positive association with termination (OR = 6.30, 95% CI [2.36, 16.82], *p* < .001). Near-miss status was not a reliable predictor of closure.

**Table 9:** Opening-level logistic regression models predicting streak termination within already established streaks.

Predictor	*B*	OR	95% CI for OR	*p*
Model 1 (parsimonious)				
Opening position (centered)	0.01	1.01	[0.92, 1.11]	0.866
Opening position^2^	0.00	1.00	[0.99, 1.01]	0.816
Reward rarity 2 (vs. 1)	−0.96	0.38	[0.16, 0.90]	0.028
Reward rarity 3 (vs. 1)	−0.35	0.70	[0.28, 1.75]	0.449
Reward rarity 4 (vs. 1)	0.08	1.08	[0.33, 3.47]	0.905
Negative valence (vs. neutral)	−0.20	0.82	[0.29, 2.30]	0.700
Positive valence (vs. neutral)	−0.10	0.90	[0.36, 2.25]	0.819
Low emotional intensity (vs. not observable)	0.64	1.91	[0.86, 4.20]	0.112
Moderate emotional intensity (vs. not observable)	0.22	1.24	[0.43, 3.53]	0.692
High emotional intensity (vs. not observable)	1.01	2.75	[0.83, 9.14]	0.099
Model 2 (exploratory)				
Opening position (centered)	0.00	1.00	[0.92, 1.10]	0.944
Opening position^2^	0.00	1.00	[0.99, 1.01]	0.999
Reward rarity 2 (vs. 1)	−0.55	0.58	[0.22, 1.49]	0.255
Reward rarity 3 (vs. 1)	−0.13	0.88	[0.34, 2.27]	0.796
Reward rarity 4 (vs. 1)	0.41	1.50	[0.42, 5.39]	0.533
Negative valence (vs. neutral)	−0.62	0.54	[0.18, 1.63]	0.275
Positive valence (vs. neutral)	−0.56	0.57	[0.21, 1.56]	0.274
Low emotional intensity (vs. not observable)	1.69	5.41	[2.10, 13.95]	< 0.001
Moderate emotional intensity (vs. not observable)	1.34	3.81	[1.20, 12.15]	0.024
High emotional intensity (vs. not observable)	2.28	9.81	[1.63, 58.95]	0.013
Near miss present (vs. no near miss)	−0.72	0.49	[0.10, 2.35]	0.370
Ambiguous/partial near miss (vs. no near miss)	0.10	1.11	[0.41, 3.03]	0.836
Reward type 1 (vs. type 2)	−0.56	0.57	[0.12, 2.72]	0.486
Reward type 3 (vs. type 2)	1.84	6.30	[2.36, 16.82]	< 0.001
Reward type 4 (vs. type 2)	0.35	1.42	[0.46, 4.41]	0.545

Outcome variable = streak termination (0 = continuation, 1 = termination). Odds ratios (OR) are reported with 95% confidence intervals. Model 1 included opening position, reward rarity, visible affective valence, and emotional intensity. Model 2 additionally included near-miss status and reward type. Standard errors were clustered by episode. Opening position was centered prior to estimation, and a quadratic term was included to test potential non-linear position effects. Model 2 should be interpreted as exploratory given the number of predictors relative to the number of termination events.

Taken together, these results suggest that streak termination may be less a matter of a single especially rare reward and more a matter of disruption in the ongoing flow of the sequence. Put differently, the sequence appeared more likely to end when the opening moment became more behaviorally marked or evaluatively salient, rather than simply because a rare reward occurred. This interpretation is consistent with the broader distinction between persistence and quitting as related but non-identical dimensions of within-session gambling behavior (9). Termination findings therefore point more clearly to interruption of flow than to rarity alone.

### Verbalizations within streaks

To address RQ4, we analyzed how streamers' verbalizations and contextual performance markers accompanied continuation and termination within loot box opening streaks. The coded qualitative corpus within streaks included 299 opening-level verbal or contextual excerpts. Of these, 243 included explicit streamer verbalizations, whereas 56 included relevant contextual or performative notes without explicit verbalization. Because some discourse functions were expressed through both speech and performance in the livestreaming context, the following descriptive analysis refers to the coded verbal/contextual corpus.

Prior gambling research has shown that verbalizations produced during gambling-like activities may include chance-related beliefs, erroneous perceptions, rationalizations, cost appraisals, and interpretive framings of outcomes (12–17). However, these statements should not be treated as direct or transparent indicators of internal cognition, because gambling-related speech may also reflect *post hoc* interpretation, task demands, self-presentation, or attempts to make sense of uncertain outcomes (14–16). In livestreamed loot box openings, this interpretive caution is especially important because verbalizations are produced in front of an audience and may therefore function simultaneously as evaluative commentary, social narration, and public performance.

**Figure 1:**
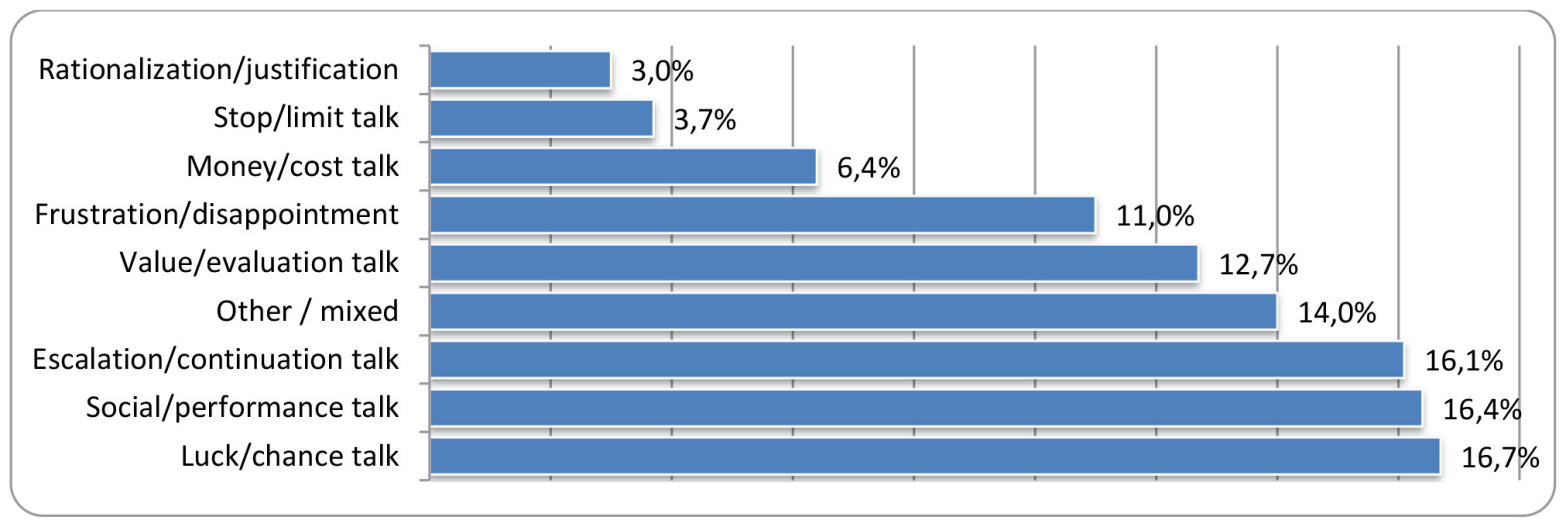
Relative frequency of the main discourse functions identified in the coded verbal/contextual corpus.

The qualitative and mixed analytic results indicated that verbalizations functioned primarily as evaluative, interpretive, and performative accompaniments to ongoing streak activity rather than as robust stopping mechanisms. At the broadest level, the verbal/contextual material observed within streaks was dominated by several recurrent functions. As shown in Table 10, the most frequent categories were luck/chance talk, social or performance-oriented talk, escalation/continuation talk, value/evaluation talk, and frustration/disappointment. Figure 1 visually summarizes the relative frequency of these discourse functions, highlighting the predominance of chance-oriented, performative, and continuation-related speech within the coded corpus.

**Table 10:** Main discourse functions identified in the coded verbal/contextual corpus within streaks.

Discourse function	*n*	%	Analytic definition
Luck/chance talk	50	16.7	Verbalizations framing the opening in terms of luck, chance, expectation, probability, or “almost” obtaining the desired item.
Social/performance talk	49	16.4	Verbalizations oriented toward the audience or shaped by the performative logic of livestreaming (e.g., addressing viewers, dramatizing the moment, inviting reaction).
Escalation/continuation talk	48	16.1	Verbal markers of continuation or renewed commitment to opening (e.g., “another one,” “let's keep going,” “one more”).
Other/mixed	42	14.0	Verbalizations that did not fit clearly into the main analytic categories or combined multiple weakly expressed functions.
Value/evaluation talk	38	12.7	Verbalizations evaluating the obtained reward in terms of usefulness, quality, desirability, or value.
Frustration/disappointment	33	11.0	Verbalizations expressing dissatisfaction, frustration, disappointment, or dissatisfaction with the obtained outcome.
Money/cost talk	19	6.4	Explicit references to cost, spending, currency, buying, or the economic burden of continued opening.
Stop/limit talk	11	3.7	Verbalizations indicating a limit, intended stopping point, or attempt to halt the sequence (e.g., “last one,” “I'm stopping”).
Rationalization/justification	9	3.0	Verbalizations justifying continued opening or explaining the meaning of the outcome in a way that supports ongoing engagement.

Percentages are calculated over the coded verbal/contextual corpus restricted to openings within streaks (*N* = 299). Discourse functions reflect the final analytic classification integrating the original verbalization categories and emergent markers identified during corpus coding.

A particularly important finding was that verbalizations did not appear to function primarily as effective interruption devices. Explicit stop or limit talk was relatively rare in the corpus (3.7%), and when it did occur it did not reliably coincide with termination. By contrast, money/cost talk, although less frequent overall (6.4%), appeared comparatively more often near closing moments than near routine continuation. Frustration/disappointment also appeared more termination-compatible than several other categories, although most instances still occurred during continuation. Meanwhile, luck/chance talk and escalation/continuation talk were overwhelmingly concentrated in openings that were followed by another opening.

Another notable pattern was the prominence of audience-oriented or performative speech. Social/performance talk represented one of the most frequent discourse categories, indicating that streamers were often not merely verbalizing internal reactions but also staging the opening for an audience, addressing viewers directly, dramatizing the moment, or embedding the sequence in a shared interactional frame. This pattern extends prior work on gambling-related verbalizations by showing how chance-related, evaluative, cost-related, and justificatory speech can be embedded within a public streaming performance (11–16). It also aligns with recent work on gambling-like elements in livestreamed environments, where visibility, audience participation, and platform-specific performance are central to how such activities are framed and experienced (11).

Table 11 provides illustrative examples of the main discourse functions, including representative quotes in the original language, English translations, and analytic interpretations.

**Table 11:** Illustrative examples of streamer verbalizations within loot box opening streaks.

Discourse function	Original quote	English translation	Analytic interpretation
Luck/chance talk	“Es 2,6%, ¿de acuerdo? (…) algún día en cuanto me gire voy a dar la vuelta y me voy a volver loco.”	“It's 2.6%, okay? (…) 1 day, when I turn around, I'm going to look back and go crazy.”	Frames the opening through probability, expectation, and anticipation of a rare outcome.
Social/performance talk	“Yo quiero un clip sin mirar, tío. ¿No molaría un clip sin mirar, gente?”	“I want a no-look clip, man. Wouldn't a no-look clip be cool, guys?”	Shows how the opening is staged for the audience and framed as streamable content.
Escalation/continuation talk	“Un premio más, el cual es… precioso.”	“One more reward, which is… beautiful.”	Marks the sequence as ongoing and normalizes continuation from one opening to the next.
Value/evaluation talk	“Este tiene que valer pasta.”	“This one has to be worth money.”	Evaluates the reward in terms of economic or exchange value.
Frustration/disappointment	“A ver… pues a mí no me sale nada.”	“Well… I'm not getting anything.”	Expresses dissatisfaction with the outcome while remaining within the opening context.
Money/cost talk	“¿Cuánto me ha costado abrir esto?”	“How much did it cost me to open this?”	Makes the monetary cost of opening explicit during the sequence.
Stop/limit talk	“Vale, última.”	“Okay, last one.”	Signals an intended limit, although such statements did not consistently coincide with actual termination.
Rationalization/justification	“Ahora me tiene que dar el cuchillo para compensar.”	“Now it has to give me the knife to compensate.”	Justifies continued opening through a compensatory expectation after previous spending or poor outcomes.

Quotes were translated for reporting purposes. Original wording was retained where possible, while minor contextual details were omitted when necessary to preserve anonymity and readability.

Overall, the qualitative findings support the interpretation that verbalizations within streaks are better understood as situated discourse accompanying an unfolding sequence than as direct stopping mechanisms. In this sense, streamer speech did not simply reveal private thoughts about loot box outcomes; it also helped frame the opening sequence as a public, evaluative, and performative activity.

## Discussion

### Main findings

The present study examined loot box opening behavior in livestreamed gaming contexts as a sequential, contextualized, temporally dense, and discursive phenomenon. Across the analyzed material, openings occurred predominantly within multi-opening streaks rather than as isolated events, and this pattern remained robust under a stricter ≥3-opening sensitivity threshold. Streak formation was more closely associated with visible monetization-related cues than with broad contextual trigger categories, and many opening-to-opening transitions occurred within short intervals. Once a streak had begun, continuation was high and was not strongly explained by reward rarity, visible valence, or near misses alone. By contrast, streak termination appeared more closely linked to disruption in the ongoing flow of the sequence than to reward rarity by itself. Finally, streamer verbalizations functioned primarily as evaluative, interpretive, and socially performative accompaniments to opening activity rather than as reliable mechanisms of self-interruption.

Taken together, these findings support a process-based account of livestreamed loot box openings. Rather than treating loot boxes only as discrete reward events, the results suggest that opening behavior in public streaming contexts may be better understood as a repeated sequence shaped by monetization salience, temporal density, behavioral momentum, and public verbal framing. This interpretation complements existing work on loot box reward salience, psychophysiological responses, associations with gaming- and gambling-related problems, and broader process-based accounts of behavioral addiction (1–3, 5–7, 10).

### Loot box openings as sequential streaks rather than isolated events

The first major contribution of the study is empirical and conceptual: loot box openings in this sample were organized predominantly into multi-opening streaks rather than isolated episodes. This finding matters because much of the existing literature has approached loot box engagement through self-report, correlational indicators, or experimental manipulations of discrete opening events (1–3, 5–7). While these approaches have shown that loot box purchasing is associated with gaming- and gambling-related problems and that rare rewards can elicit arousal, perceived reward value, and urge to continue opening, they do not by themselves establish how opening behavior is structured in ecologically valid public contexts (2, 3, 5, 6).

The present findings suggest that, at least in livestreamed environments, the isolated opening is often not the most informative behavioral unit. Instead, openings appear embedded within repeated sequences that develop their own internal dynamics. This aligns conceptually with sequential observational approaches, which emphasize that transitions between coded events can be analytically meaningful in their own right (19, 20), as well as with work on within-session persistence and chasing in gambling, which has shown that continued engagement within a session is not reducible to a single act or outcome, but may reflect a broader pattern of sequential involvement (9). The present study extends that insight to livestreamed loot box activity by showing that repeated opening behavior is not merely incidental, but structurally central to the observed phenomenon.

This reframing is important because it shifts the object of analysis. If loot box openings are usually embedded in streaks, then the relevant question is not only what happens during a single opening, but how an opening sequence begins, how it is maintained, and how it eventually comes to an end. In this sense, the streak provides a useful analytic unit for studying loot box behavior as an unfolding process rather than as a collection of isolated reward events.

### Monetization cues and streak formation

A second key finding concerns streak formation. Broad contextual trigger categories did not strongly or consistently explain whether an opening episode became a streak. However, more specific visible monetization-related cues, particularly temporary offers and virtual currency, were more clearly associated with the formation of multi-opening streaks. This result is theoretically meaningful because it suggests that the transition from a single opening to repeated opening activity may be shaped less by broad contextual labels and more by the immediate visual salience of the monetization environment.

In this respect, the findings complement prior work emphasizing the gambling-like and commercially engineered features of loot boxes. Research has repeatedly shown that paid loot box engagement is associated with problem gambling indicators, and that reward variability, rare outcomes, and monetization design may heighten their risky potential (1–7). The present study adds to this literature by suggesting that, in livestreamed settings, visible monetization cues may play a role not simply in whether loot boxes are available, but in whether opening behavior unfolds as a repeated sequence.

This interpretation should remain cautious. The observed cues should not be treated as direct causal mechanisms in a strong experimental sense. Rather, they are best understood as proximal and observable features of the environment that were associated with the onset of repeated opening behavior. Even so, the finding has implications for public health and platform governance, because it suggests that repeated opening activity may be facilitated not only by the reward structure of loot boxes themselves, but also by the interface contexts in which those openings are visually embedded (1, 4, 7, 8).

### Continuation within streaks as momentum rather than reward spikes

A third important finding is that once a streak had begun, continuation was the dominant outcome. Most openings within streaks were followed by another opening, indicating that continuation, rather than immediate stopping, was the typical pattern within established sequences. This supports the view that streaks may acquire a degree of sequential inertia once underway.

At the same time, the analyses did not support a simple explanation in which this continuity was primarily driven by reward rarity, visible positive valence, or near misses. This is especially notable because rarity and near-miss-like events have been central to how loot boxes are theorized as gambling-like mechanics. Experimental loot box studies have shown that rare rewards can increase arousal, reward responses, and urge to continue opening, suggesting that individual outcomes can have motivational salience (2, 3). The present findings do not contradict that literature. Rather, they suggest that in livestreamed contexts, event-level reward impact and sequence-level continuation may be related but non-identical processes.

This interpretation is consistent with process-based accounts of behavioral addiction, which emphasize that repeated behavior may emerge through the interaction of reward learning, environmental cues, affective states, habits, and situational opportunities rather than through isolated reinforcing events alone (10). In the present study, continuation appeared less like a series of independent decisions following each outcome and more like the persistence of an already established opening sequence. The language of momentum should therefore be understood descriptively and analytically, not causally: it captures the observed tendency for openings to continue once a streak had begun, without implying that a single mechanism fully explains that continuation.

### Streak termination as disruption of flow

The termination findings complement this interpretation. If continuation within streaks was common and not strongly explained by rare rewards or near misses, termination appeared to occur when the ongoing flow of opening became more behaviorally marked or evaluatively salient. Visible emotional intensity and some reward outcome categories were more closely associated with closure than reward rarity alone. This suggests that ending a streak may involve an interruption in the sequence rather than simply the arrival of a sufficiently rare or satisfying outcome.

This distinction between continuation and termination is important. In many analyses of loot boxes, the key question is whether a given outcome increases the urge to open more boxes. That question remains valuable, especially in experimental settings. However, the present findings suggest that, in ecological livestreamed contexts, the decision-like moment of stopping may be analytically different from the process of continuing. Continuation may be sustained by the already active sequence, whereas termination may require some form of disruption, marking, or reorientation of attention.

This interpretation aligns with broader gambling research suggesting that persistence and quitting are related but distinct within-session processes (9). It also supports the broader argument that loot box openings should not be studied only through isolated reward effects. Understanding repeated opening requires attention to the temporal and behavioral organization of the sequence, including the moments at which that sequence loses momentum or becomes interrupted.

### Verbalizations as situated and performative discourse

The qualitative findings extend this argument by clarifying how verbalizations operate within livestreamed loot box openings. The dominant discourse functions in the corpus were evaluative, interpretive, and performative rather than clearly self-regulatory. Luck/chance talk, escalation markers, social/performance talk, and value/evaluation talk were common, whereas explicit stop or limit talk was comparatively rare and did not reliably coincide with termination.

This finding is consistent with earlier work on gambling-related verbalizations, which has shown that spoken statements during gambling-like tasks may reveal cognitive distortions, evaluations, or chance-related beliefs, but should not be treated as direct and transparent indicators of internal cognition (12–17). The present study extends that caution to livestreaming contexts, where verbalizations are not only cognitive or interpretive, but also publicly staged. In these environments, streamers are not simply talking through an experience; they are often narrating, dramatizing, and socially framing it for an audience.

This helps explain why stop/limit talk did not function as a robust interruption mechanism. In a public and performative setting, speech may serve to sustain momentum, manage audience engagement, justify continued opening, or mark the event as entertaining rather than to regulate it effectively. The prominence of social/performance talk in the corpus is particularly important here and aligns with recent work showing that gambling-like elements in game streaming are shaped by visibility, audience participation, and platform-specific performance dynamics (11). Verbalizations in this context are therefore better understood as situated discourse accompanying an unfolding sequence than as straightforward windows into private cognition.

### Public health, platform governance, and regulatory implications

These findings have broader implications for how loot boxes are conceptualized from a public health perspective. Much of the debate has focused on whether loot boxes are gambling-like because of their reliance on chance and their association with problem gambling indicators (1, 7, 8). The present results suggest that another important layer deserves attention: loot box openings may operate as repeated, publicly staged sequences supported by visible monetization cues and sustained through momentum-like dynamics rather than isolated reward events alone.

This has implications for platform design and governance. If visible currency indicators, temporary offers, and similar interface cues are associated with the formation of repeated opening streaks, then these features may warrant scrutiny not only as monetization devices but also as facilitators of sustained engagement. Likewise, if livestreamed openings are publicly dramatized and socially reinforced, then the potential influence of streaming platforms may extend beyond simple exposure to include the visibility, normalization, and performative amplification of gambling-like behavior (8, 11).

The results do not justify strong causal claims about harm from observational data alone. However, they do suggest that public health discussions of loot boxes may benefit from considering the interaction between game mechanics, interface salience, temporal density, and platformed performance, rather than focusing exclusively on single opening events or individual psychological reactions. From a regulatory perspective, this supports the need for clearer oversight of products that occupy an ambiguous position between gaming and gambling. Such oversight should not be restricted to the internal mechanics of the games themselves, but should also take into account the broader digital environments in which loot box content is circulated, staged, and amplified.

At the same time, the present findings should not be interpreted as direct evidence for specific intervention strategies. The study examined publicly streamed opening behavior, not individual player trajectories, family contexts, adolescent outcomes, or clinical patterns of problematic use. Therefore, implications for prevention and early intervention should be framed as hypotheses for future research rather than as direct recommendations. Future studies should examine whether similar sequential and performative dynamics are present in private play, adolescent samples, family contexts, or gacha-based mobile games, and whether interventions aimed at reducing exposure to monetization cues or disrupting repetitive opening routines have measurable preventive value.

## Strengths and limitations

This study has several strengths. It examined loot box openings in ecologically valid, publicly accessible livestream environments rather than relying solely on retrospective self-report or tightly controlled experimental tasks. It also combined quantitative and qualitative approaches, allowing the phenomenon to be analyzed as both a sequential behavioral process and a discursive one. In addition, inter-rater agreement was high across the central coded variables, supporting the reliability of the observational framework used in the analyses.

At the same time, several limitations should be acknowledged. First, the sample was a purposive convenience sample drawn from a relatively small number of streams, which limits generalizability. Second, the study focused on publicly available VOD material, meaning that interpretation was constrained by what was visible and audible in the archived streams. Third, although the coding framework captured a range of contextual, affective, temporal, and discourse-related variables, some constructs, particularly those related to motivation, subjective experience, strategic intent, or audience reception, could only be inferred indirectly and were therefore not treated as primary explanatory variables. Fourth, the qualitative component was intended as a focused content analysis of opening-related verbal/contextual excerpts rather than a full discourse analysis or ethnography of streamer communities. Finally, because the study was observational, the results should not be interpreted causally. The identified associations help characterize the organization and framing of loot box opening behavior, but they do not establish causal mechanisms.

## Future directions

Future research could extend this work in several ways. First, larger and more diverse samples of livestreams would help determine how robust these patterns are across games, platforms, streamer profiles, audience sizes, and linguistic contexts. Second, future studies could examine whether different monetization interfaces, reward systems, or audience conditions alter the probability of streak formation, continuation, termination, or temporal density. Third, mixed-methods work combining observational coding with experimental or psychophysiological designs could help clarify how event-level reward salience interacts with sequence-level momentum (2, 3). Fourth, more detailed study of audience interaction may help determine whether chat activity, viewer prompts, or performance incentives shape the maintenance of loot box streaks in ways not fully captured here.

Finally, future research should examine whether the patterns observed in livestreamed contexts also appear in private play and among adolescents or young adults. This is especially important given prior evidence linking loot box purchasing with Internet Gaming Disorder, Online Gambling Disorder, and later online gambling involvement in younger samples (5, 6). Such work could help determine whether streak-like opening sequences are primarily a feature of public performance, a broader behavioral pattern of loot box engagement, or an interaction between monetization design, social visibility, and individual vulnerability.

## Concluding remarks

The present study provides an ecological and sequential account of loot box opening behavior in livestreamed gaming contexts. Across the analyzed material, openings were organized predominantly as multi-opening streaks rather than isolated events. Streak formation was associated more strongly with visible monetization-related cues than with broad contextual trigger categories, while continuation within streaks appeared better characterized by sequential momentum than by rare rewards, visible positive reactions, or near misses alone. Termination, in turn, appeared more closely related to interruptions in the ongoing flow of the sequence. Streamer verbalizations further framed opening activity as evaluative, interpretive, and performative public discourse rather than as reliable self-interruption.

These findings suggest that loot boxes should be understood not only as chance-based reward mechanisms, but also as temporally organized and socially mediated practices that unfold within specific digital environments. From a public health and regulatory perspective, this highlights the importance of looking beyond isolated opening events to consider how monetization cues, interface design, streaming platforms, and audience-oriented performance may contribute to the visibility and persistence of gambling-like behavior in contemporary gaming.

## Data Availability

The datasets presented in this study can be found in online repositories. The names of the repository/repositories and accession number(s) can be found in the article/Supplementary material.
